# Supplementary material for: New Pyrazolo-Benzimidazole Mannich Bases with Antimicrobial and Antibiofilm Activities
Source: Antibiotics (Basel). 2022 Aug 12;11(8):1094. doi: 10.3390/antibiotics11081094 (PMC9405415; doi:10.3390/antibiotics11081094)

N-[(*1H*-3,5-dimethylpyrazol-1-yl)methyl]1-amino-*1H*-benzimidazole ***(5a)***

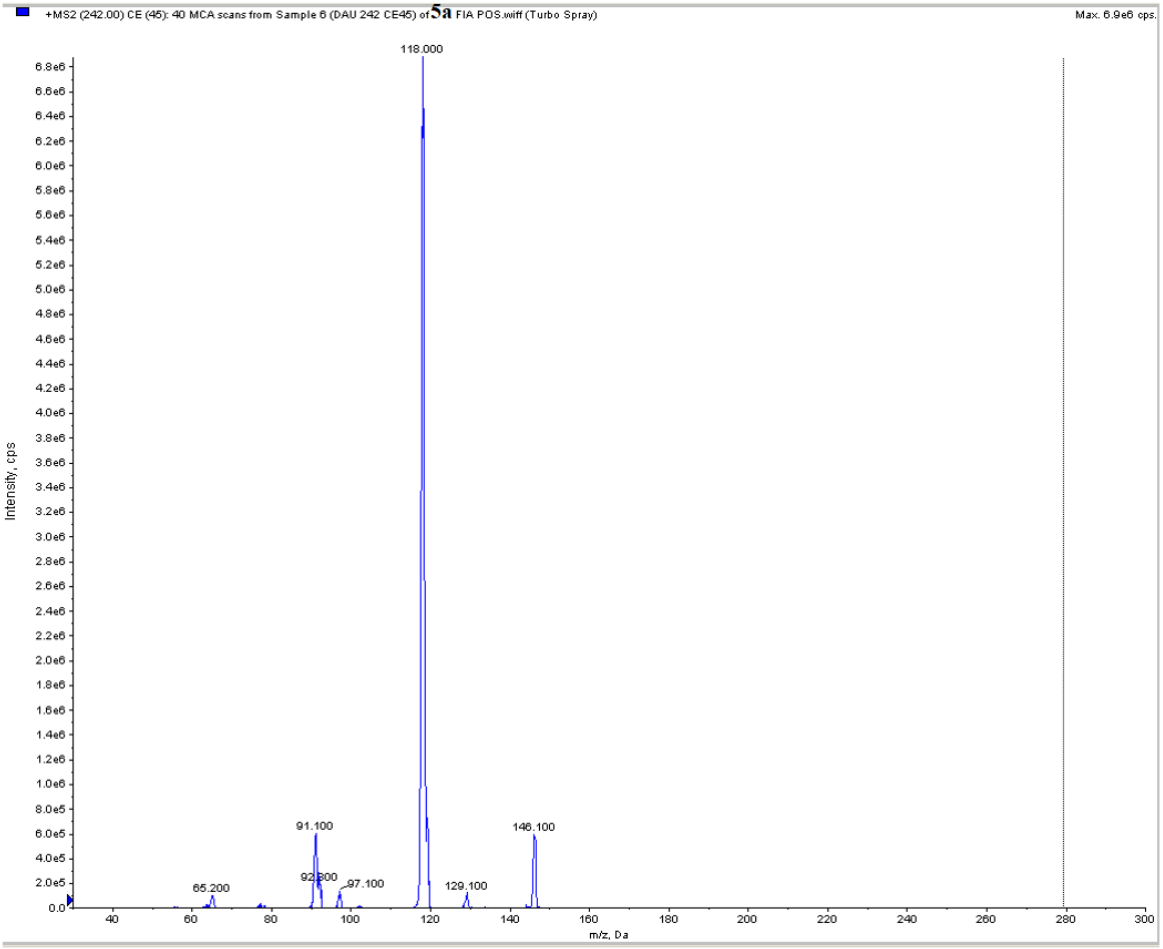

N-[(*1H*-3,5-dimethyl-4-nitropyrazol-1-yl)methyl]1-amino-*1H*-benzimidazole **(5b)**

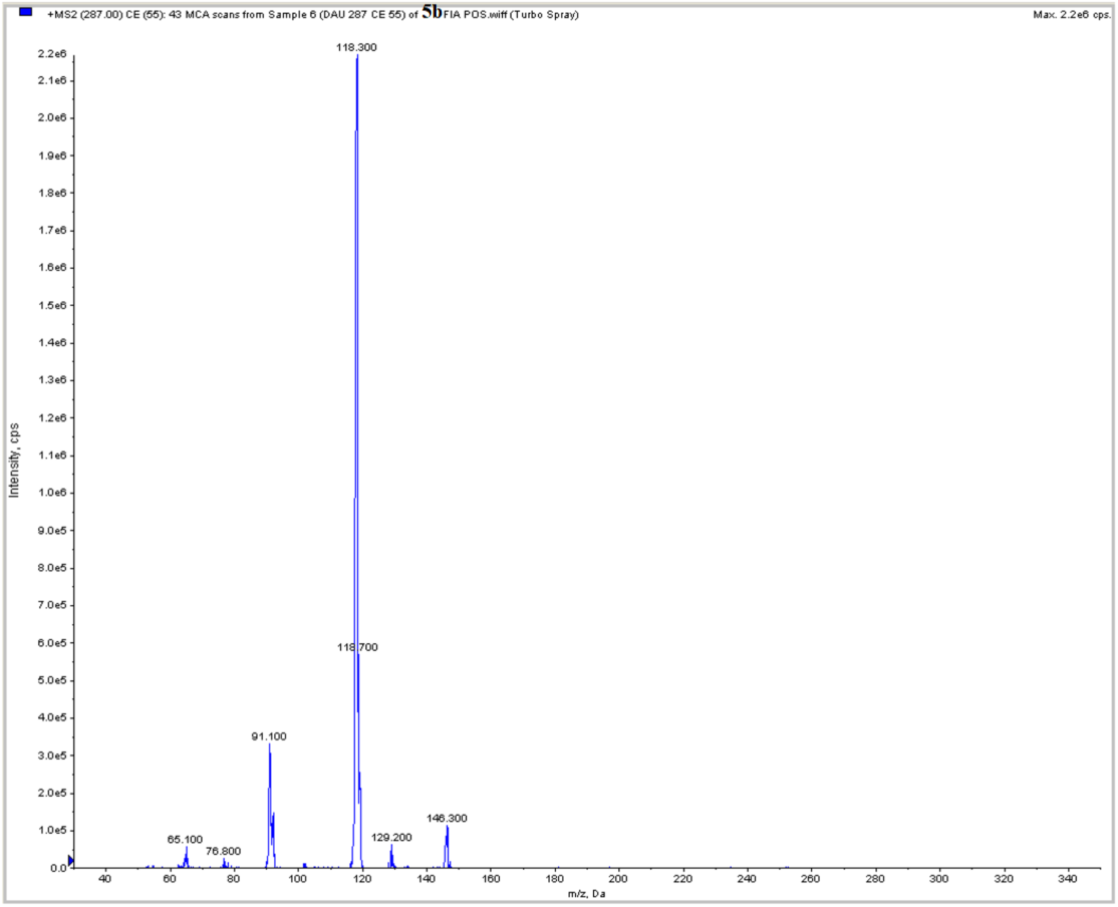

N-[(*1H*-3,5-dimethyl-4-iodopyrazol-1yl)methyl]1-amino-*1H*-benzimidazole ***(5c)***

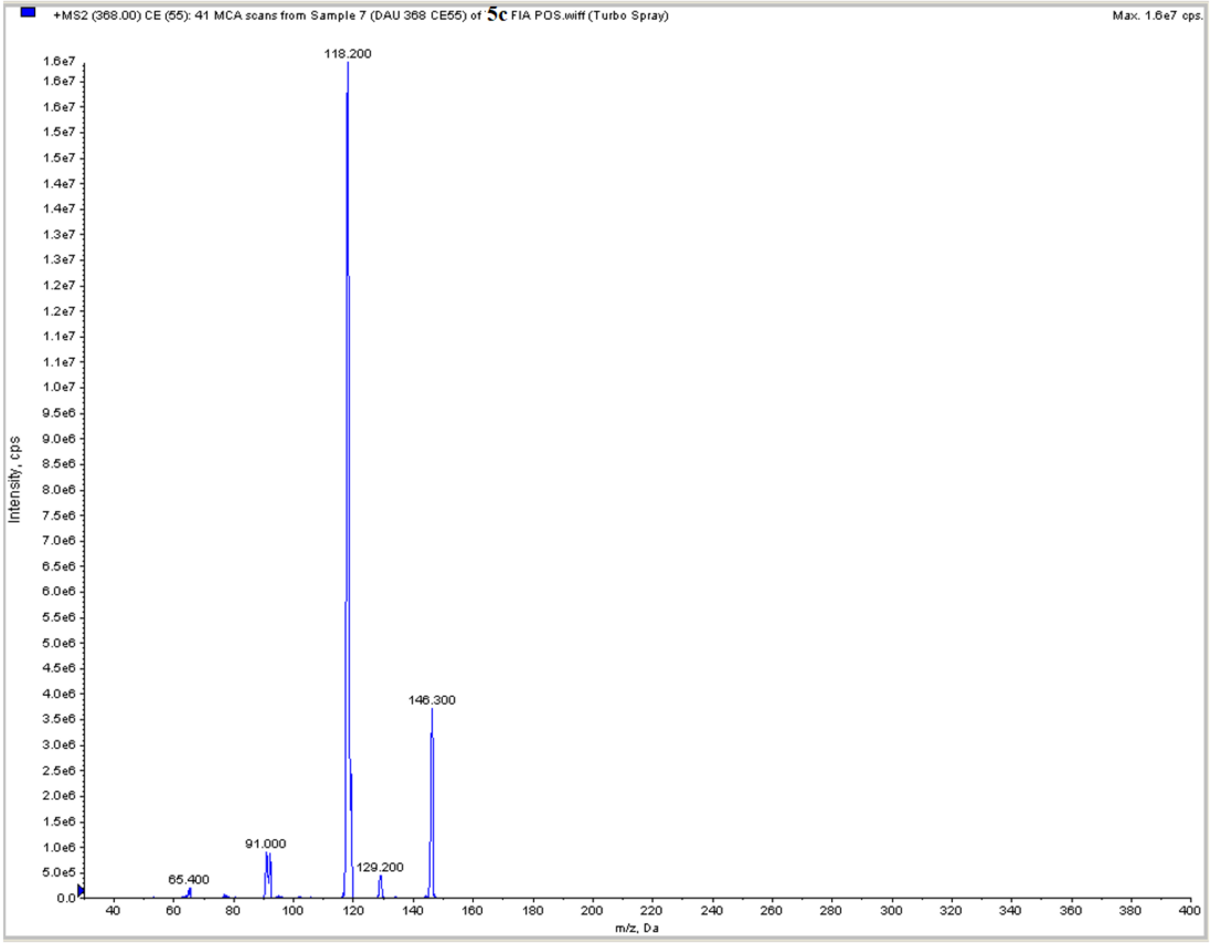

N-[(*1H*-pyrazol-1yl)methyl]1-amino-2-methyl-*1H*-benzimidazole ***(5d)***

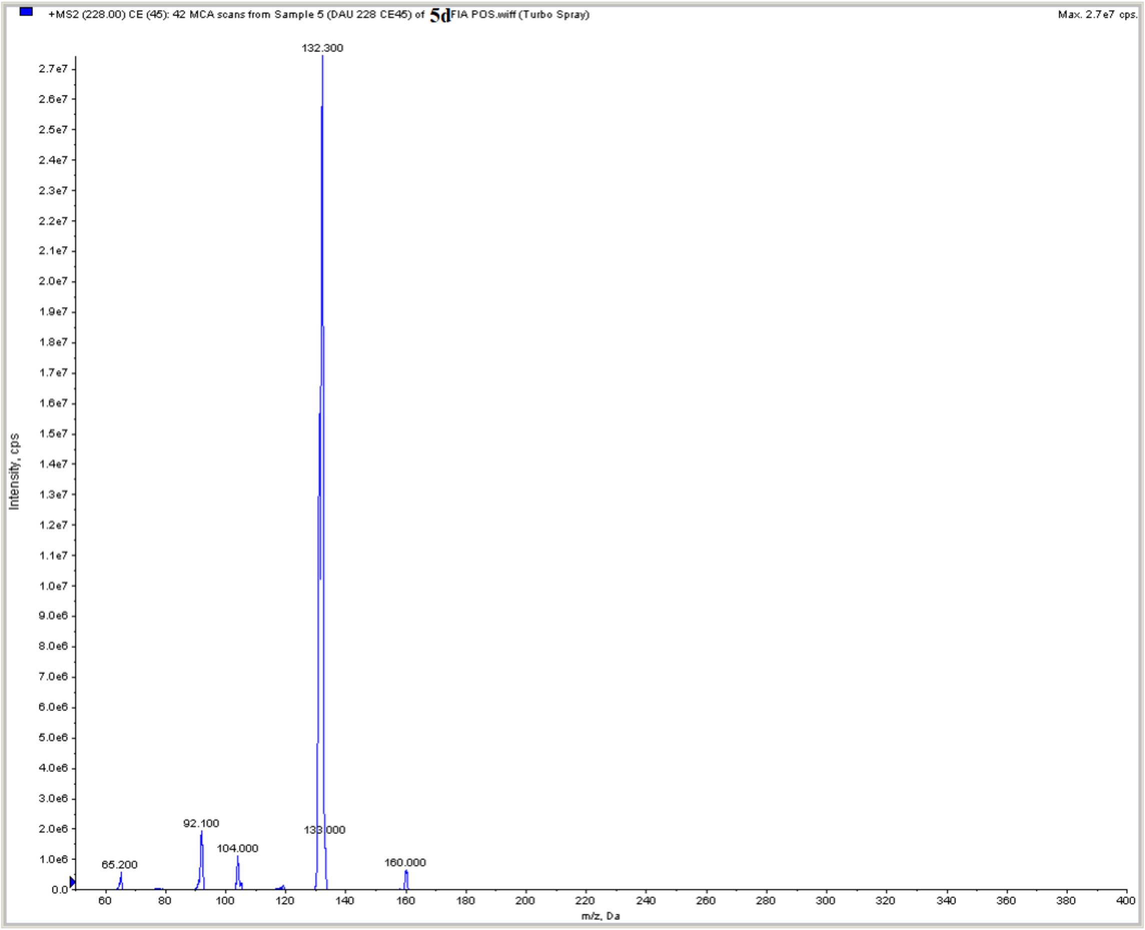

N-[(*1H*-3,5-dimetyl-pyrazol-1-yl)methyl]-1-amino-2-methyl-*1H*-benzimidazole ***(5e)***

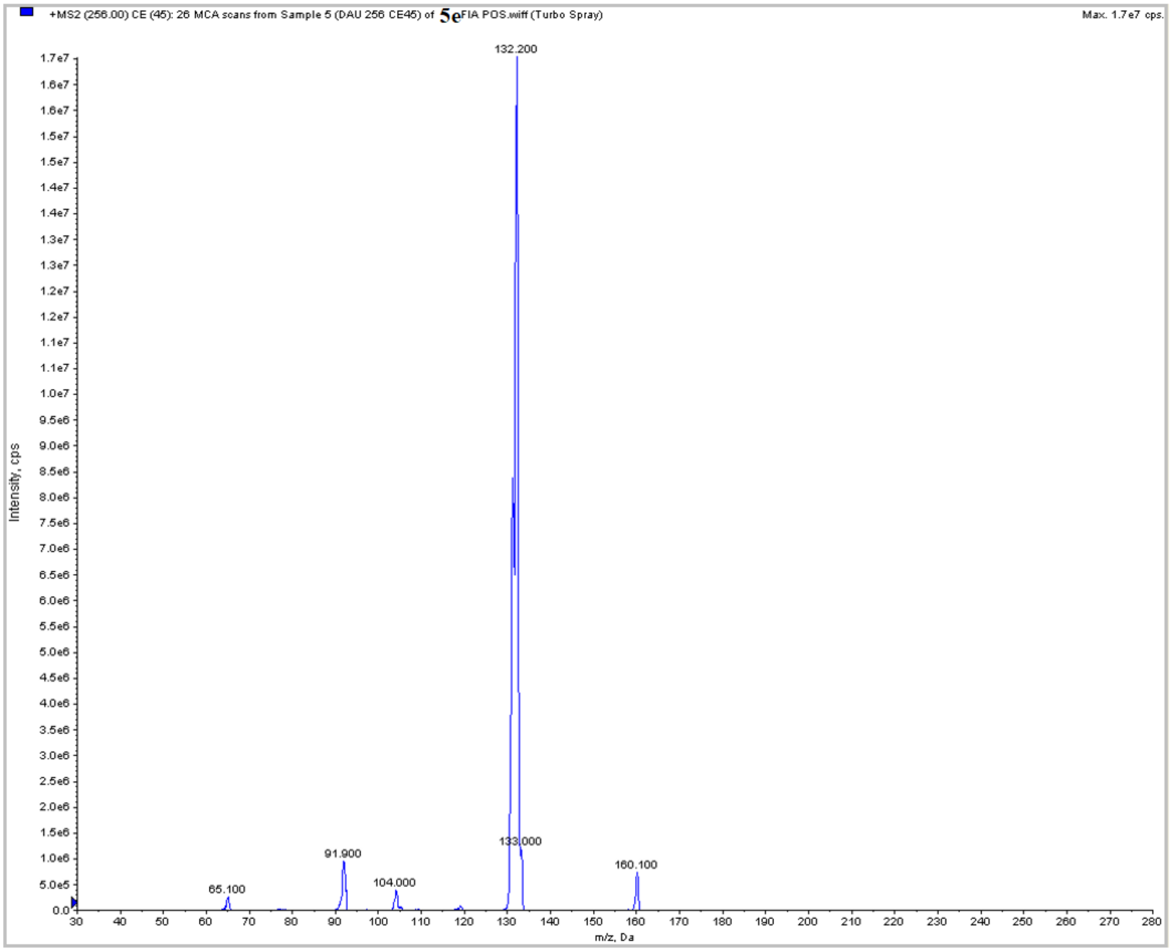

N-[(*1H*-3,5-dimethyl-4-nitropyrazol-1-yl)methyl]-1-amino-2-methyl-*1H*-benzimidazole ***(5f)***

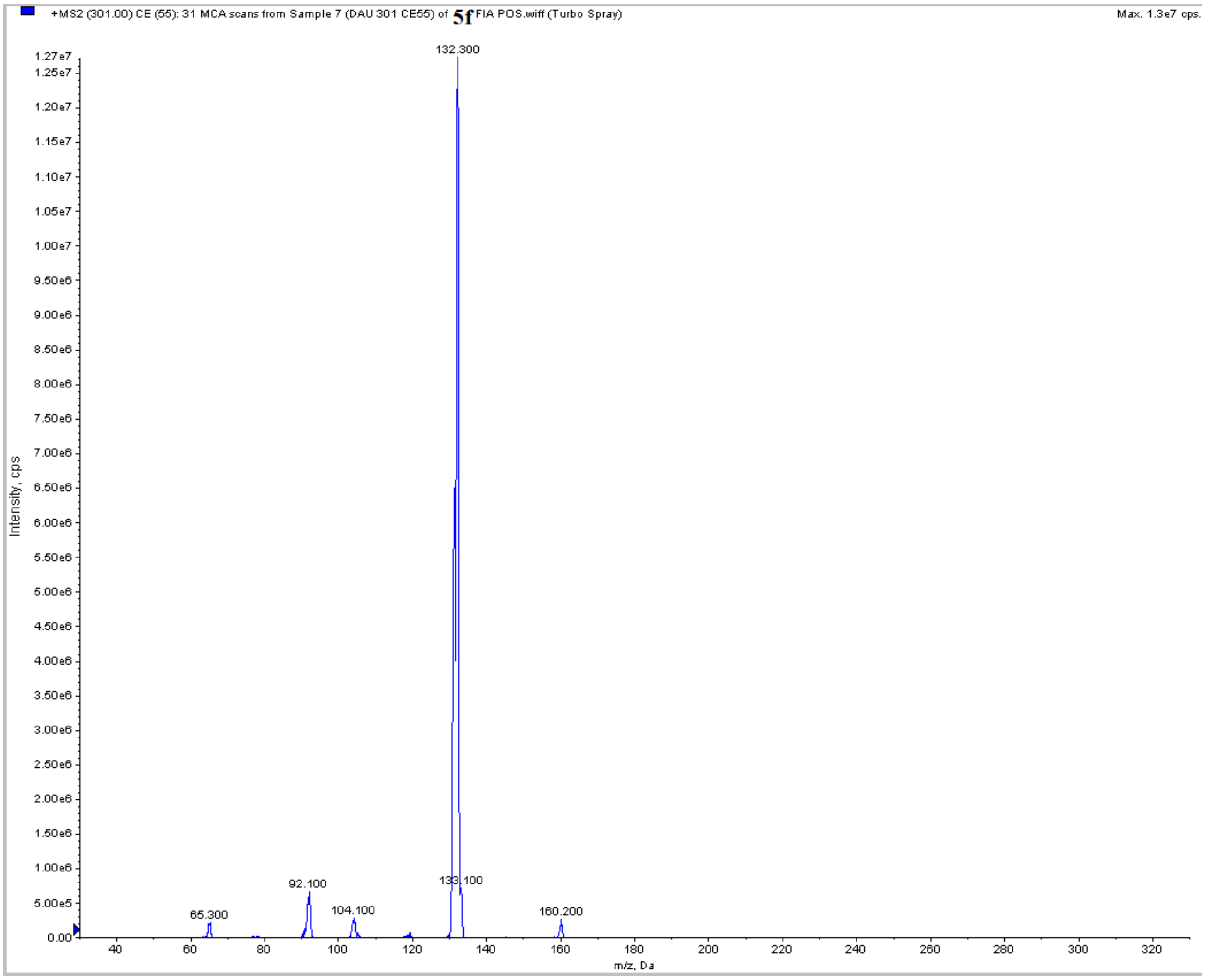

N-[(*1H*-3,5-dimethyl-4-iodopyrazol-1yl)methyl]-1-amino-2-methyl-*1H*-benzimidazole ***(5g)***

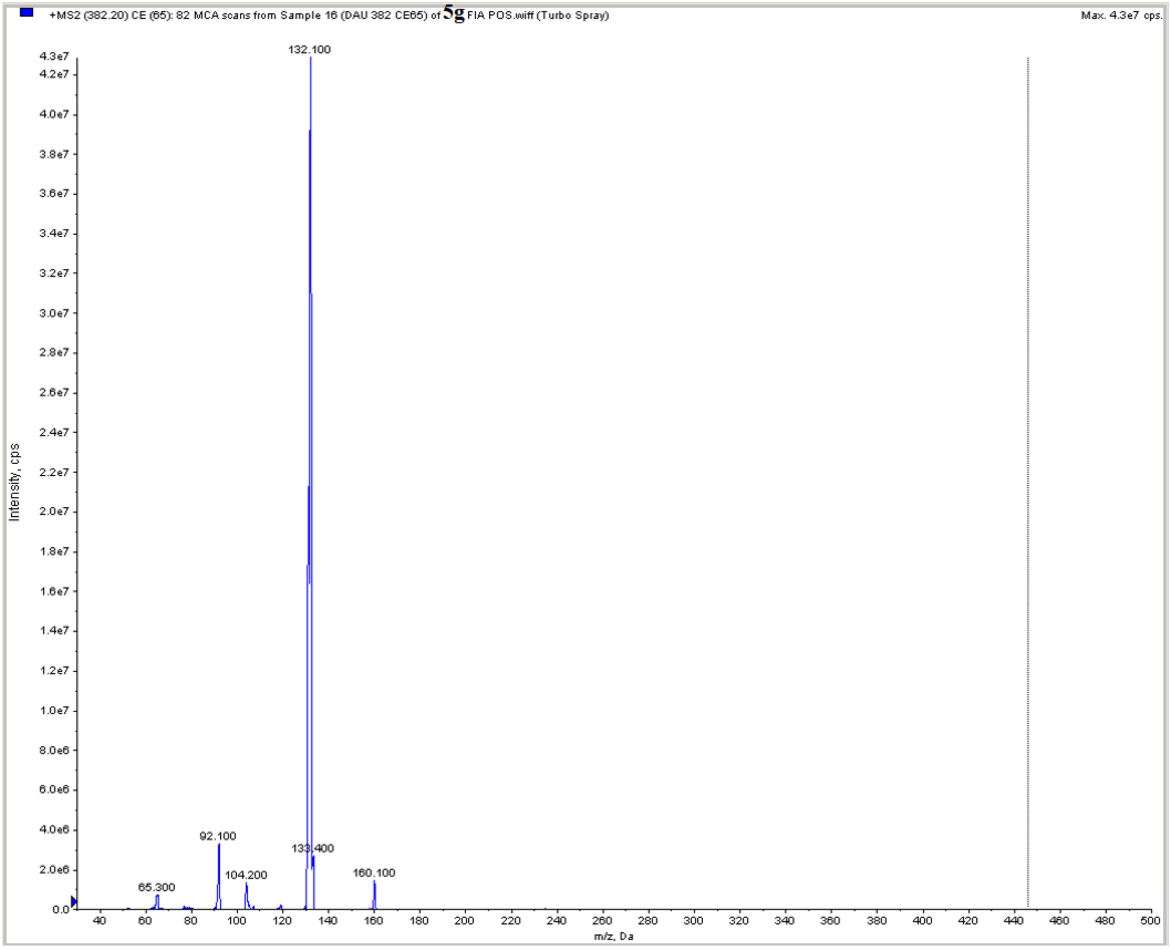

Instrument Bruker FOURIER 300 MHz (UnivBucuresti)
User Ch. Zalaru
Operator CS AM
Registry No. 4953
Sample Changer No. 6
Sample Name 5a-Z87
@H1-DUL-01 CDCl3 {D:\CCO\TCaproiu} ICON-NMR-Lab 6

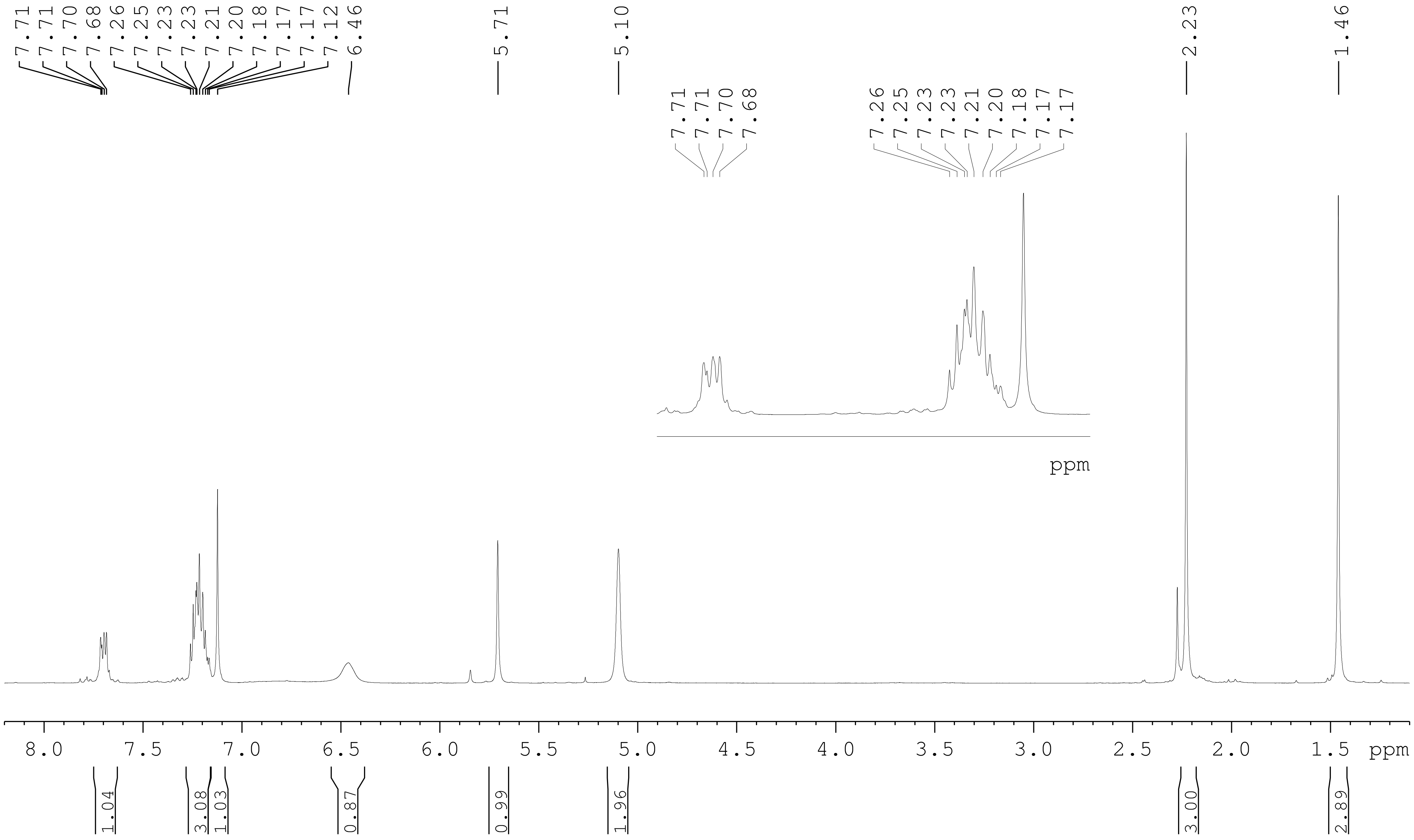

Instrument Bruker FOURIER 300 MHz (UnivBucuresti)
User Ch. Zalaru
Operator CS AM
Registry No. 4953
Sample Changer No. 6
Sample Name 5a-Z87
@C13-CPD-DUL-01 CDCl3 {D:\CCO\TCaproiu} ICON-NMR-Lab 6

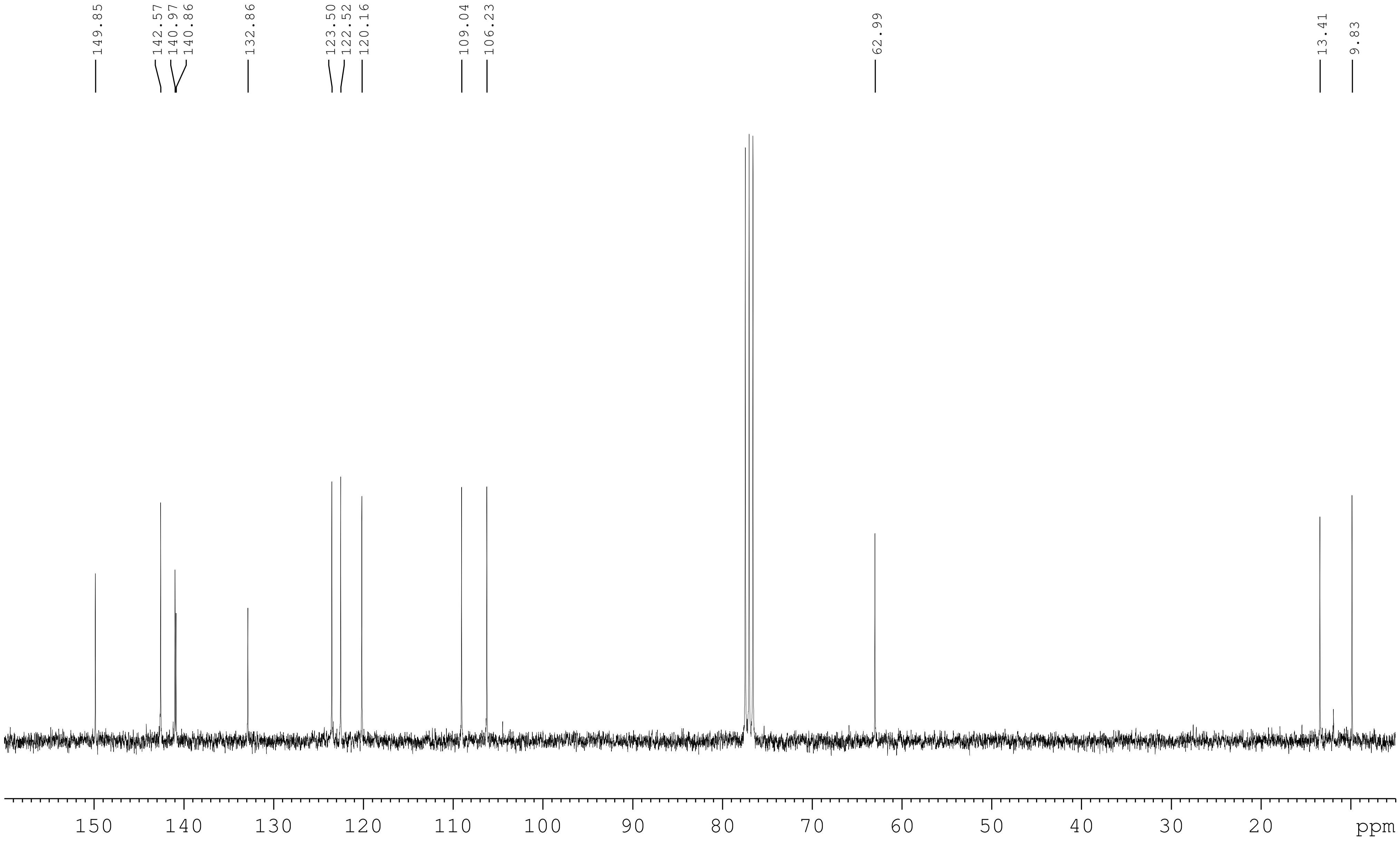

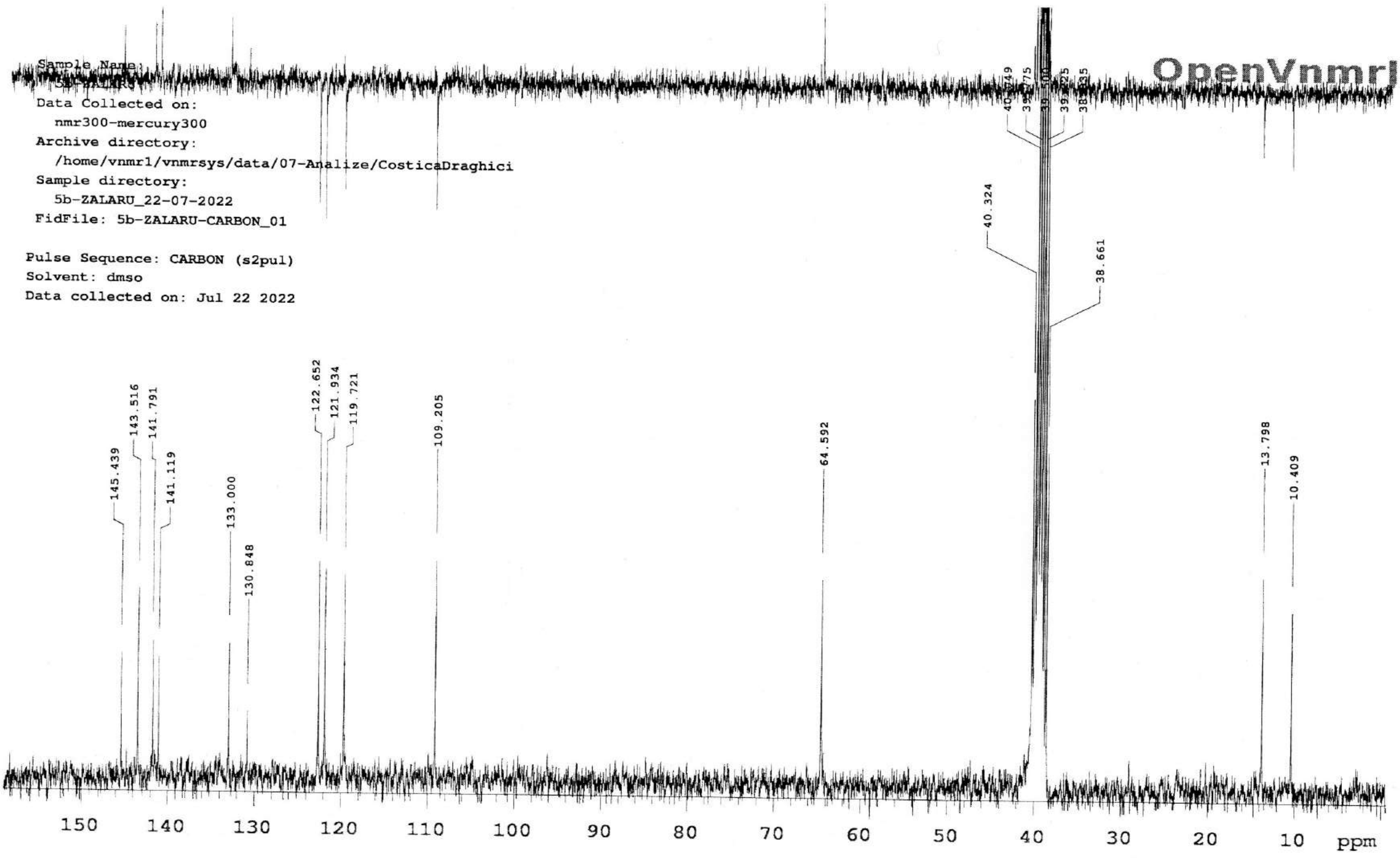
Sample Name:
Data Collected on:
nmr300-mercury300
Archive directory:
/home/vnmr1/vnmrsys/data/07-Analize/CosticaDraghici
Sample directory:
5b-ZALARU_22-07-2022
FidFile: 5b-ZALARU-CARBON_01
Pulse Sequence: CARBON (s2pul)
Solvent: dmso
Data collected on: Jul 22 2022
OpenVnmrJ
145.439
143.516
141.791
141.119
133.000
130.848
122.652
121.934
119.721
109.205
64.592
40.324
38.661
13.798
10.409
150 140 130 120 110 100 90 80 70 60 50 40 30 20 10 ppm

Sample Name:
5b-ZALARU
Data Collected on:
nmr300-mercury300
Archive directory:

Sample directory:

FidFile: PROTON

Pulse Sequence: PROTON (s2pul)
Solvent: dmso
Data collected on: Jul 22 2022

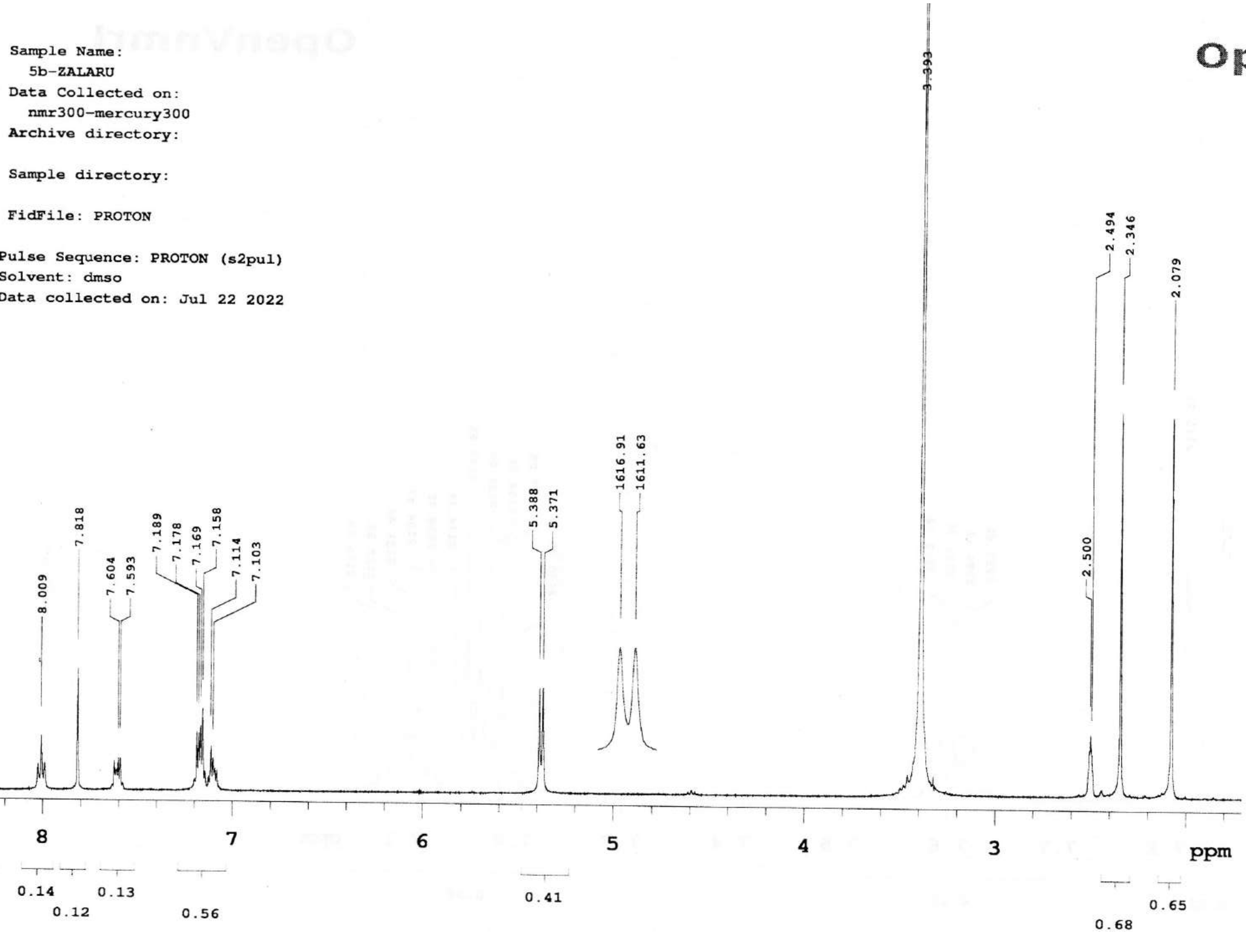

OpenVnmrJ

Sample Name:
5b-ZALARU
Data Collected on:
nmr300-mercury300
Archive directory:

Sample directory:

FidFile: PROTON

Pulse Sequence: PROTON (s2pul)
Solvent: dmso
Data collected on: Jul 22 2022

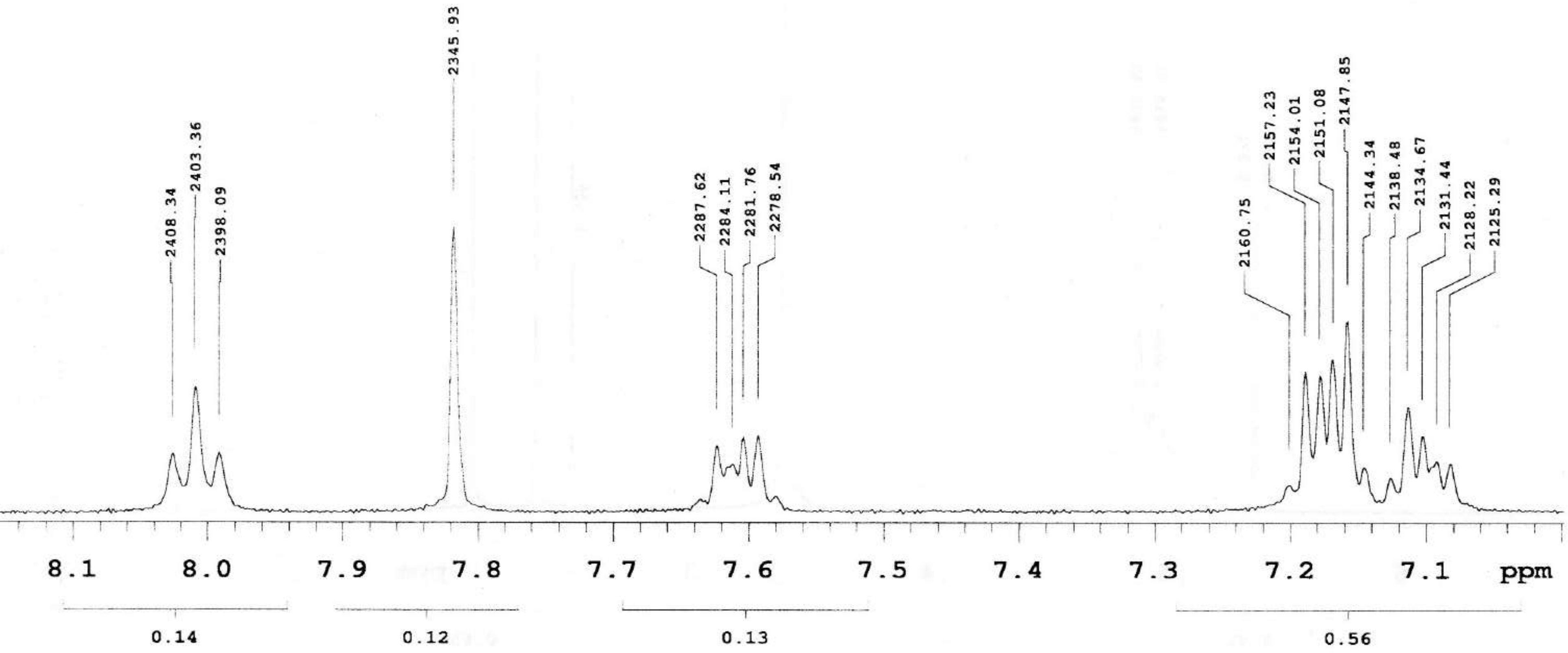

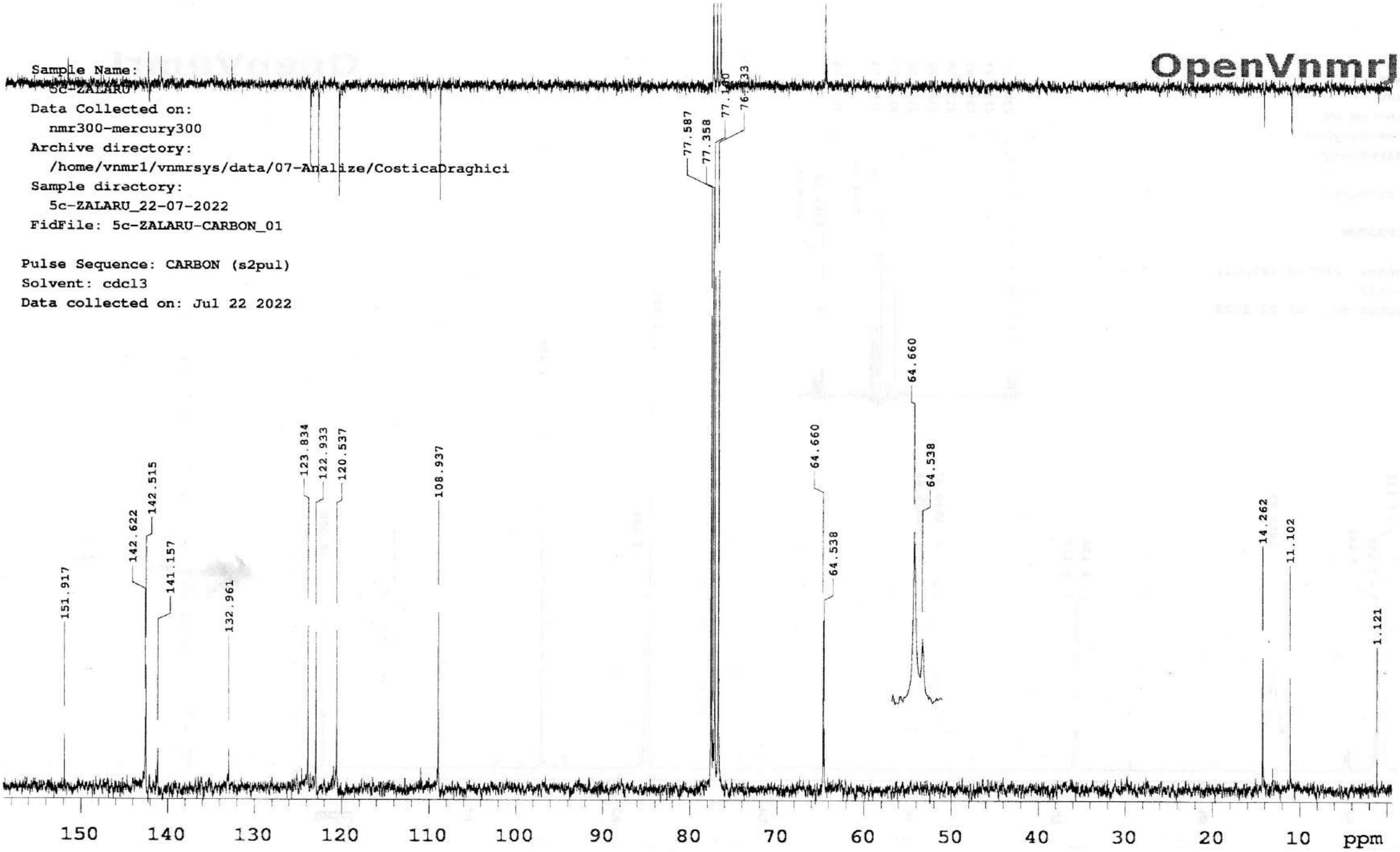
Sample Name:
5c-ZALARU
Data Collected on:
nmr300-mercury300
Archive directory:
/home/vnmr1/vnmrsys/data/07-Analize/CosticaDraghici
Sample directory:
5c-ZALARU_22-07-2022
FidFile: 5c-ZALARU-CARBON_01
Pulse Sequence: CARBON (s2pul)
Solvent: cdcl3
Data collected on: Jul 22 2022
OpenVnmrJ
151.917
142.622
142.515
141.157
132.961
123.834
122.933
120.537
108.937
77.587
77.358
64.660
64.538
64.660
64.538
14.262
11.102
1.121
150
140
130
120
110
100
90
80
70
60
50
40
30
20
10
ppm

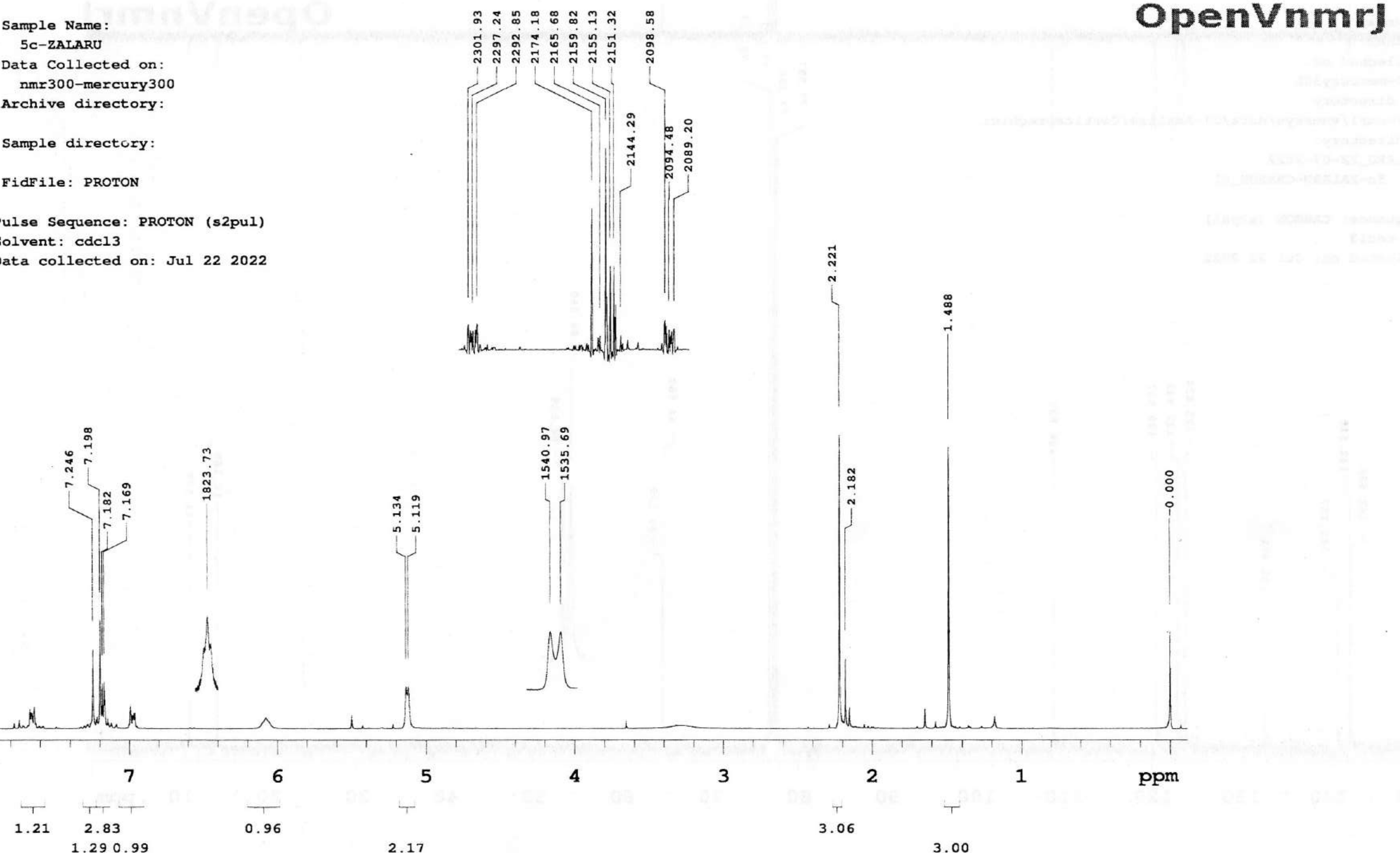
Sample Name:
5c-ZALARU
Data Collected on:
nmr300-mercury300
Archive directory:
Sample directory:
FidFile: PROTON
Pulse Sequence: PROTON (s2pul)
Solvent: cdcl3
Data collected on: Jul 22 2022
OpenVnmrJ
2301.93
2297.24
2292.85
2174.18
2165.68
2159.82
2155.13
2151.32
2098.58
2144.29
2094.48
2089.20
7.246
7.198
7.182
7.169
1823.73
5.134
5.119
1540.97
1535.69
2.221
2.182
1.488
0.000
7
6
5
4
3
2
1
ppm
1.21
2.83
1.29 0.99
0.96
2.17
3.06
3.00

^{1}H-NMR spectra

N-[(*1H*-pyrazol-1yl)methyl]-1-amino-2-methyl-*1H*-benzimidazole ***(5d)***

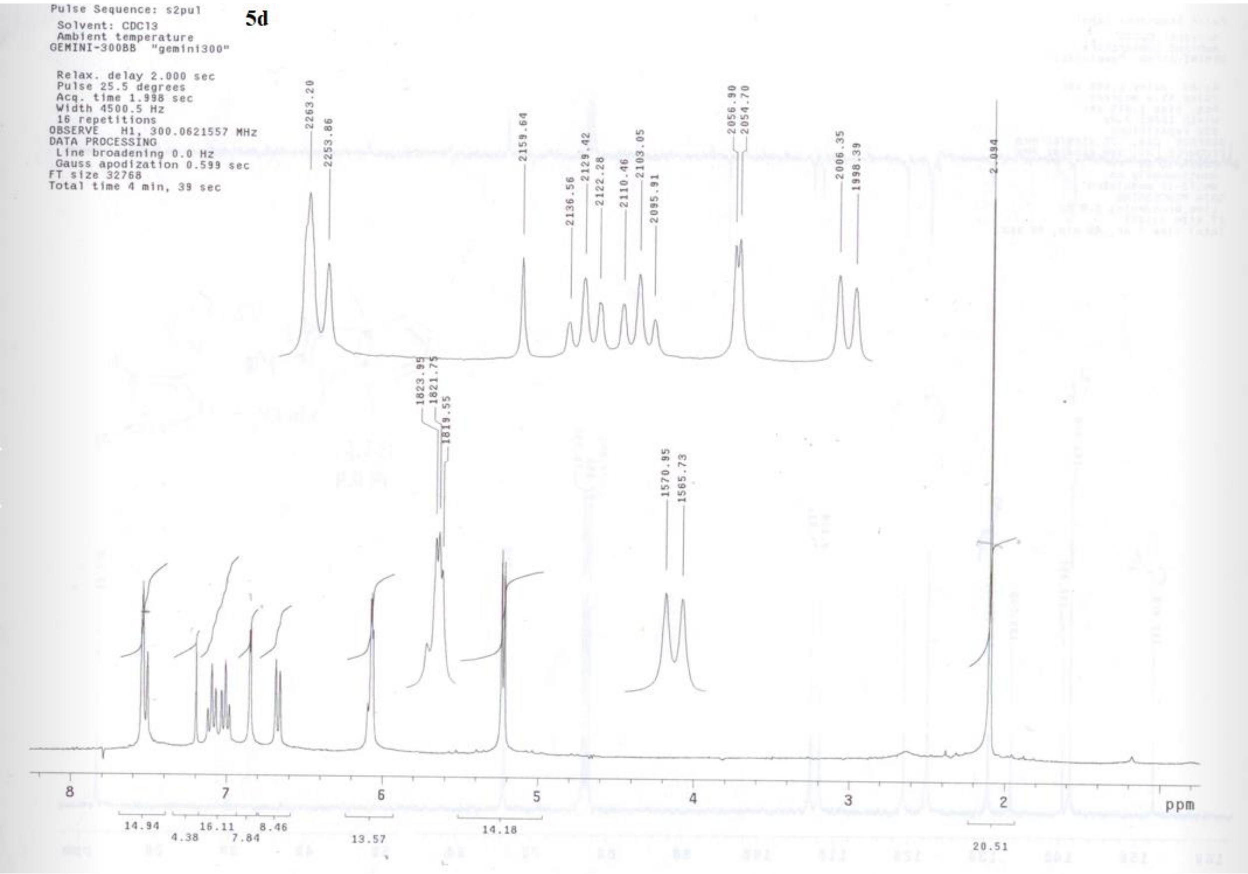

^{1}H-NMR spectra

N-[(*1H*-pyrazol-1yl)methyl]1-amino-2-methyl-*1H*-benzimidazole ***(5d)***

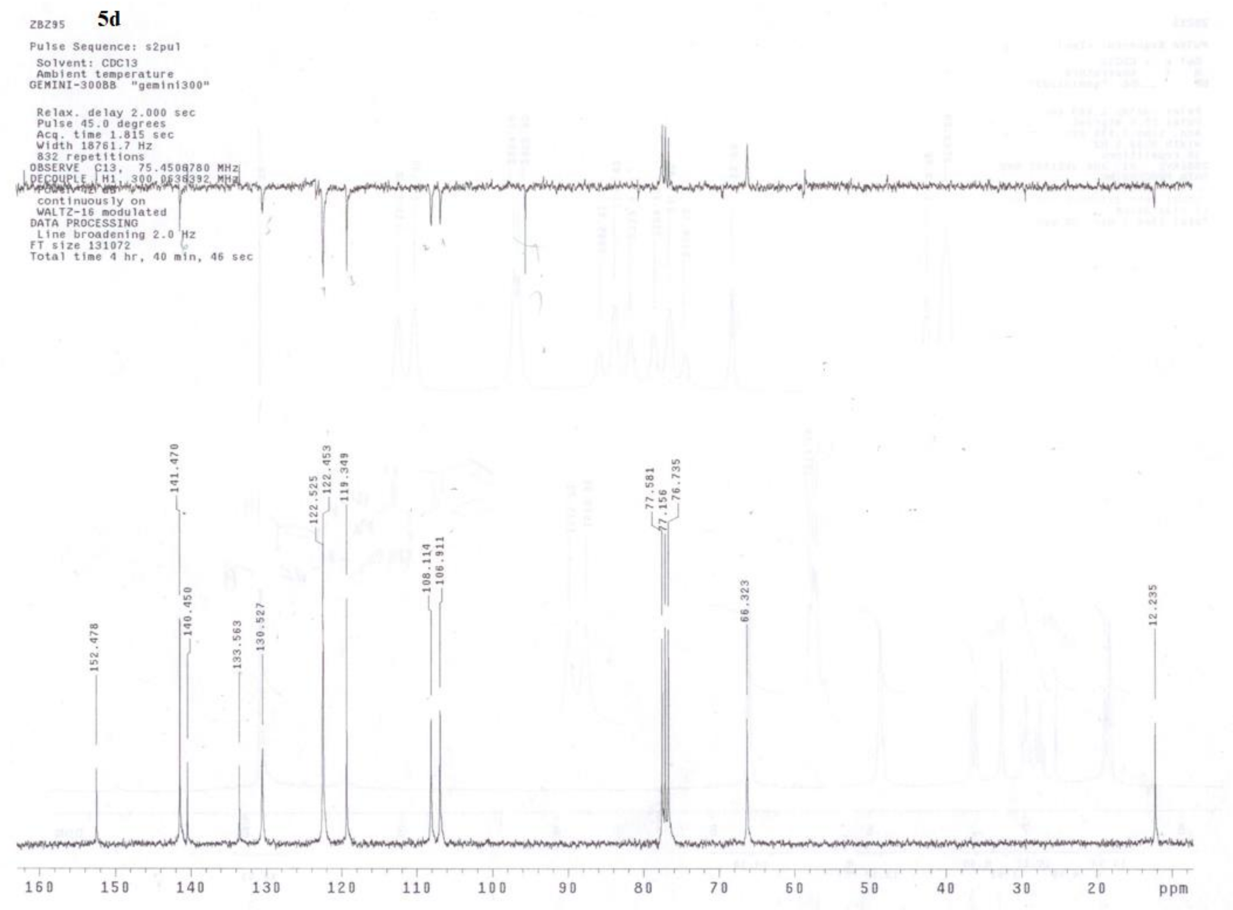

5e

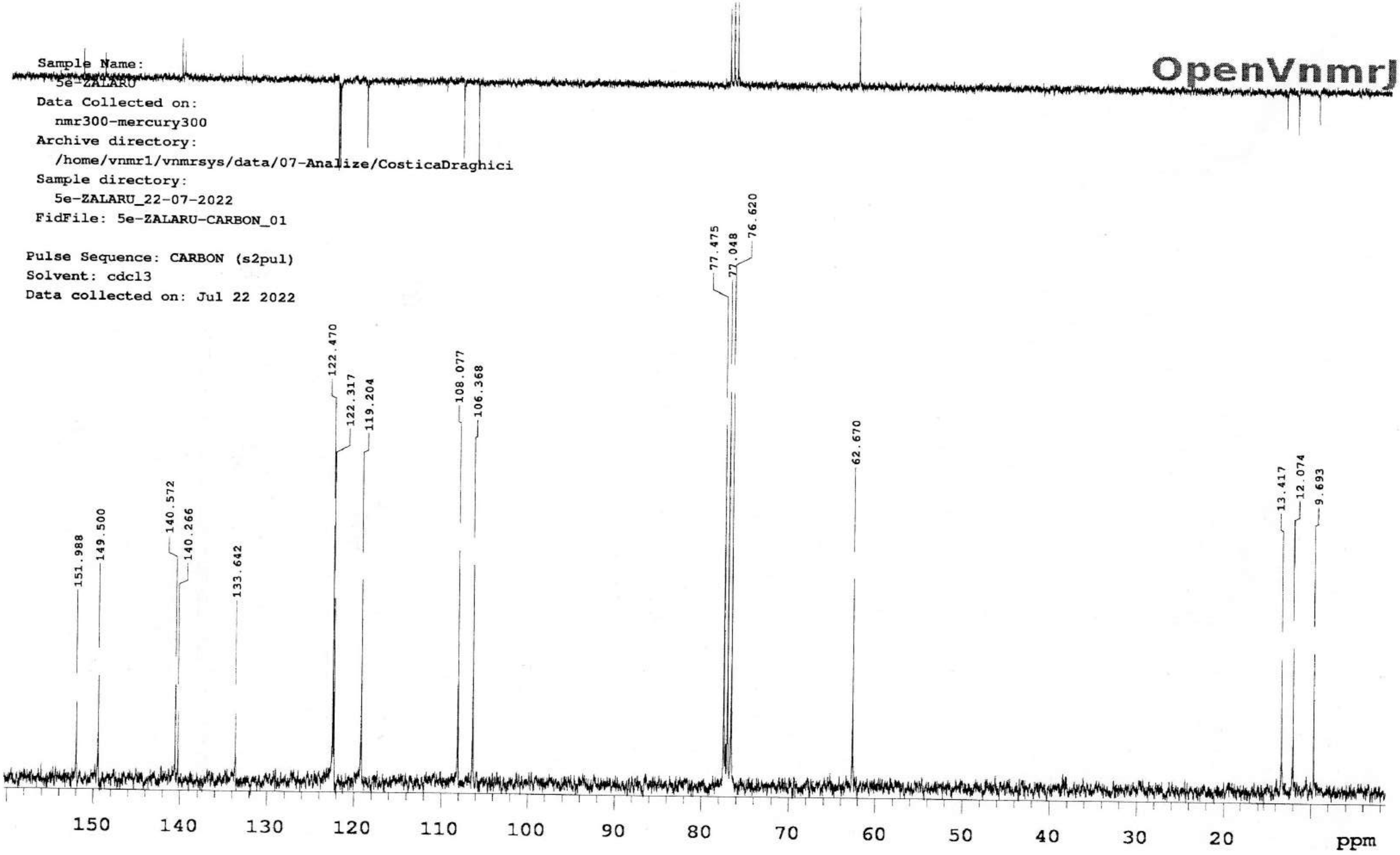
Sample Name:
5e-ZALARU
Data Collected on:
nmr300-mercury300
Archive directory:
/home/vnmr1/vnmrsys/data/07-Analize/CosticaDraghici
Sample directory:
5e-ZALARU_22-07-2022
FidFile: 5e-ZALARU-CARBON_01
Pulse Sequence: CARBON (s2pul)
Solvent: cdcl3
Data collected on: Jul 22 2022
OpenVnmrJ
151.988
149.500
140.572
140.266
133.642
122.470
122.317
119.204
108.077
106.368
77.475
77.048
76.620
62.670
13.417
12.074
9.693
150
140
130
120
110
100
90
80
70
60
50
40
30
20
ppm

Sample Name:
5e-ZALARU
Data Collected on:
nmr300-mercury300
Archive directory:

Sample directory:

FidFile: PROTON

Pulse Sequence: PROTON (s2pul)
Solvent: cdcl3
Data collected on: Jul 22 2022

OpenVnmrJ

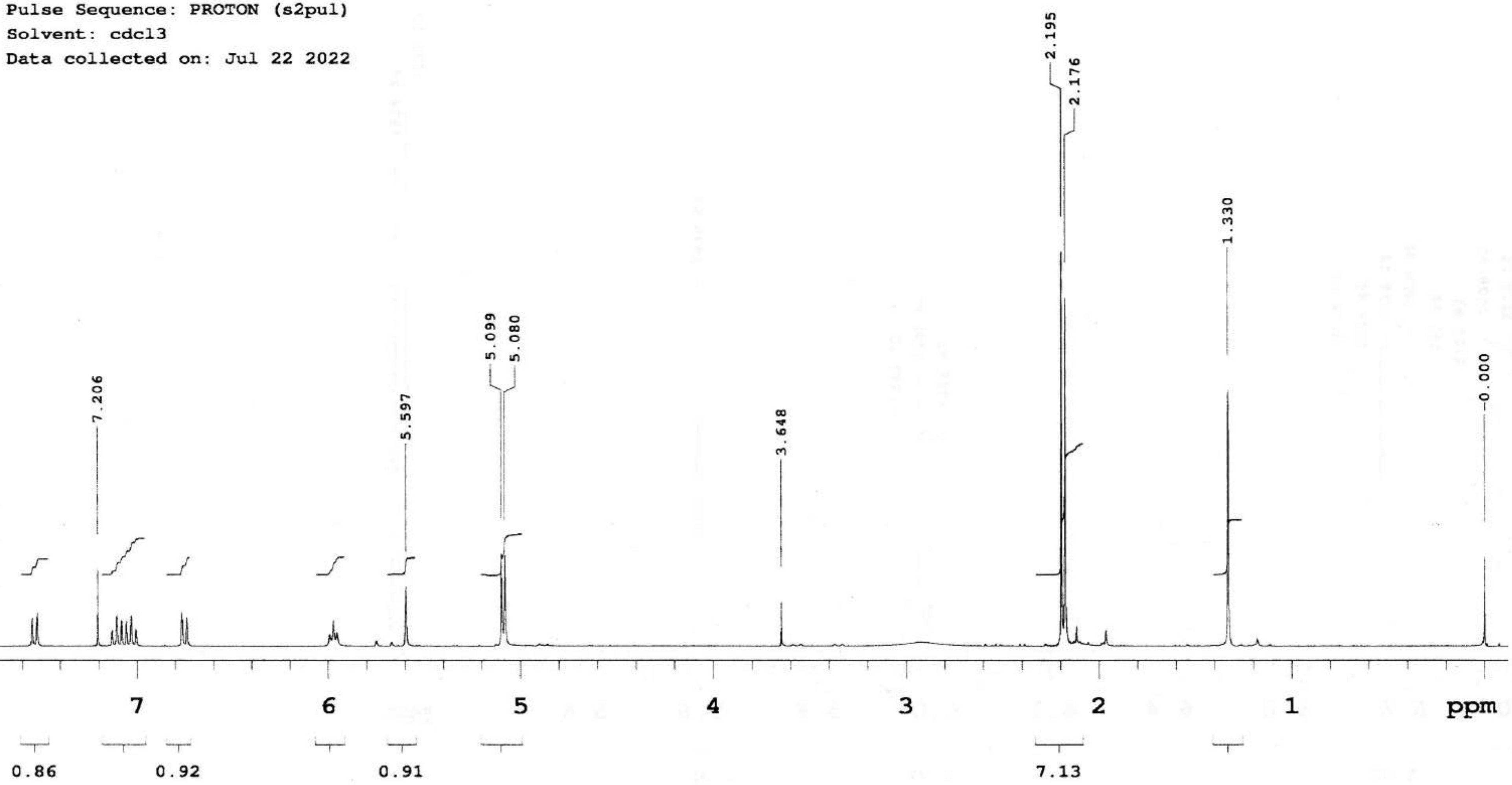

Sample Name:
5e-ZALARU
Data Collected on:
nmr300-mercury300
Archive directory:
/home/vnmr1/vnmrsys/data/07-Analize/CosticaDraghici
Sample directory:
5e-ZALARU_22-07-2022
FidFile: 5e-ZALARU-PROTON_01

Pulse Sequence: PROTON (s2pul)
Solvent: cdcl3
Data collected on: Jul 22 2022

OpenVnmrJ

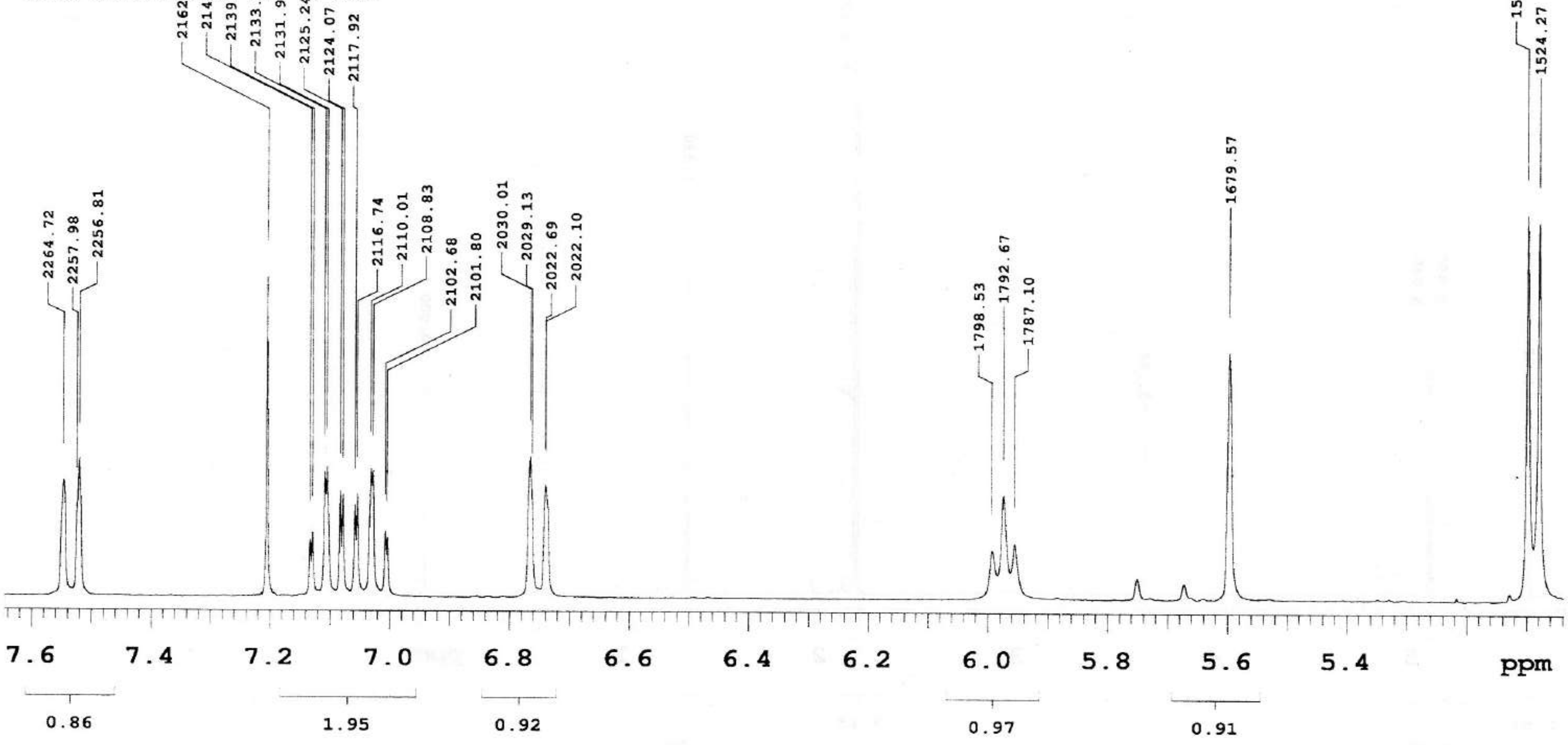

^{1}H-NMR spectra

N-[(*1H*-3,5-dimethyl-4-nitropyrazol-1-yl)methyl]-1-amino-2-methyl-*1H*-benzimidazole ***(5f)***

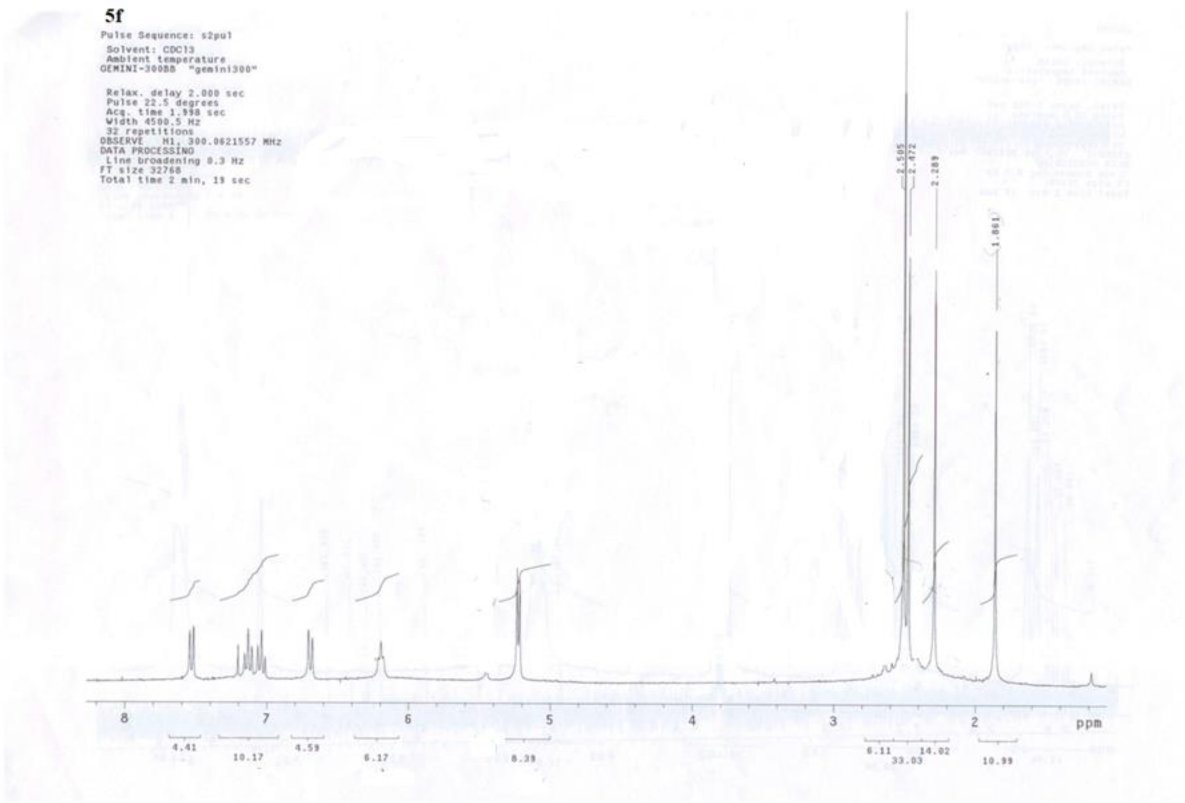

^{13}C-NMR spectra

N-[(*1H*-3,5-dimethyl-4-nitropyrazol-1-yl)methyl]-1-amino-2-methyl-*1H*-benzimidazole ***(5f)***

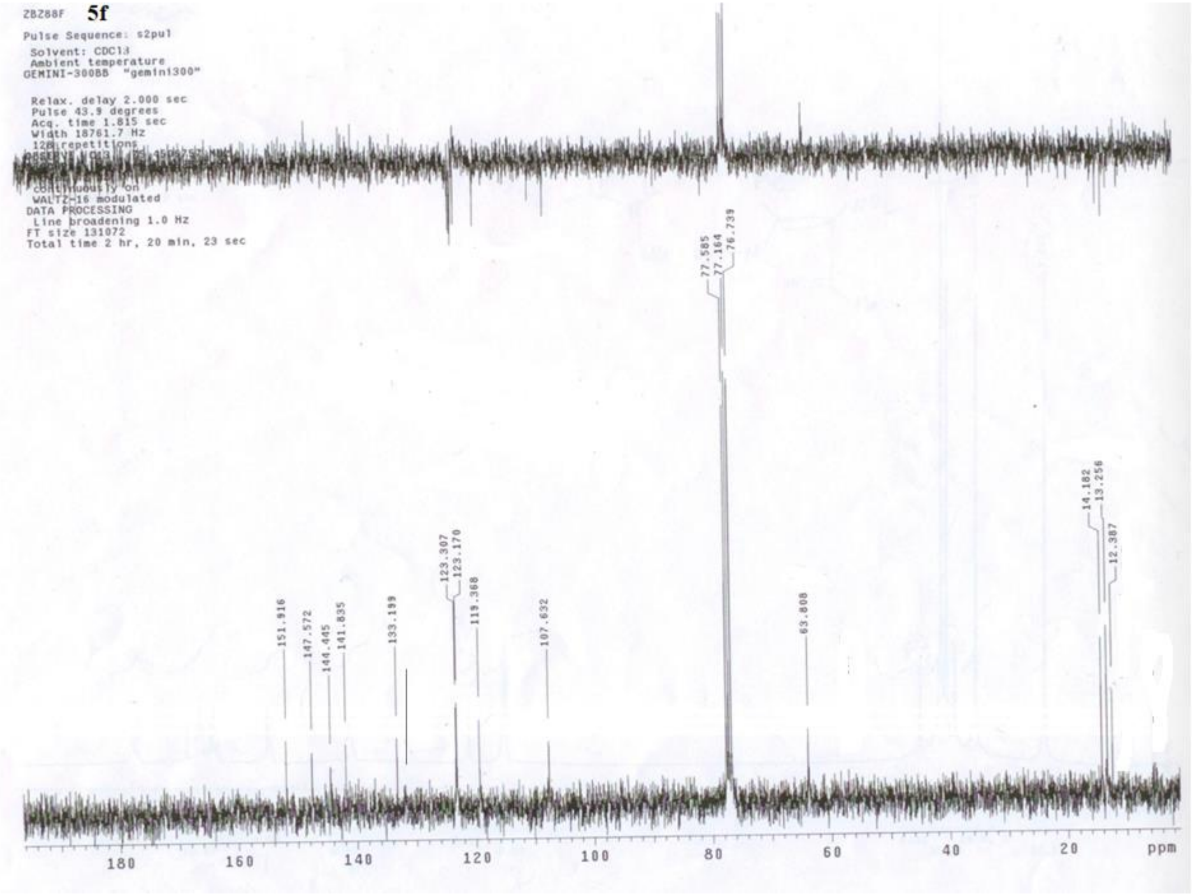

5g

Sample Name:
JEXP10
Data Collected on:
nmr300-mercury300
Archive directory:
/home/vnmr1/vnmrsys/data/07-Analize/CosticaDraghici
Sample directory:
5g-ZALARUWFTDS_22-07-2022
FidFile: 5g-ZALARUWFTDS-CARBON_01

Pulse Sequence: CARBON (s2pul)
Solvent: cdcl3
Data collected on: Jul 22 2022

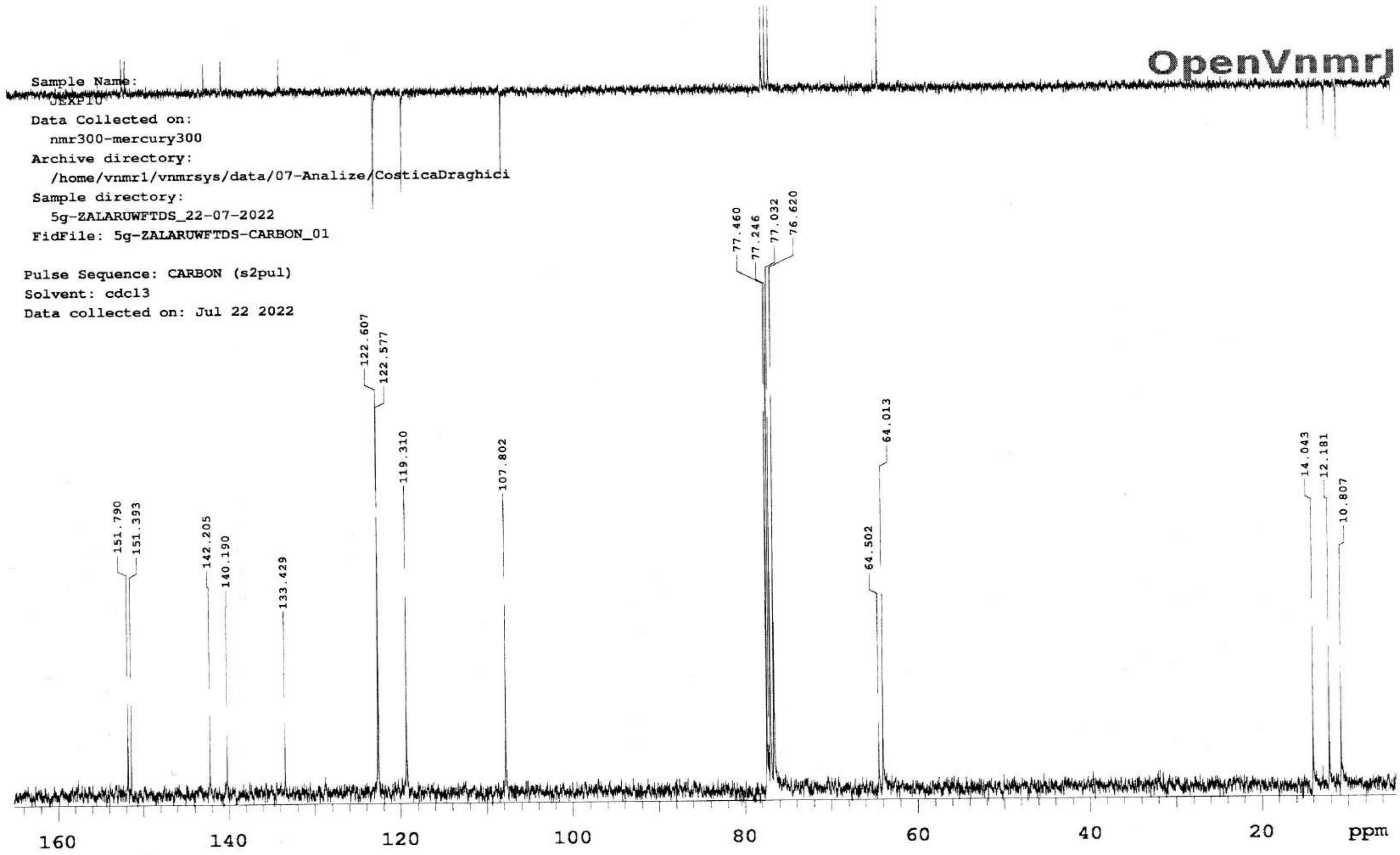

Sample Name:
5g-ZALARU
Data Collected on:
nmr300-mercury300
Archive directory:

Sample directory:

FidFile: PROTON

Pulse Sequence: PROTON (s2pul)
Solvent: cdcl3
Data collected on: Jul 22 2022

OpenVnmrJ

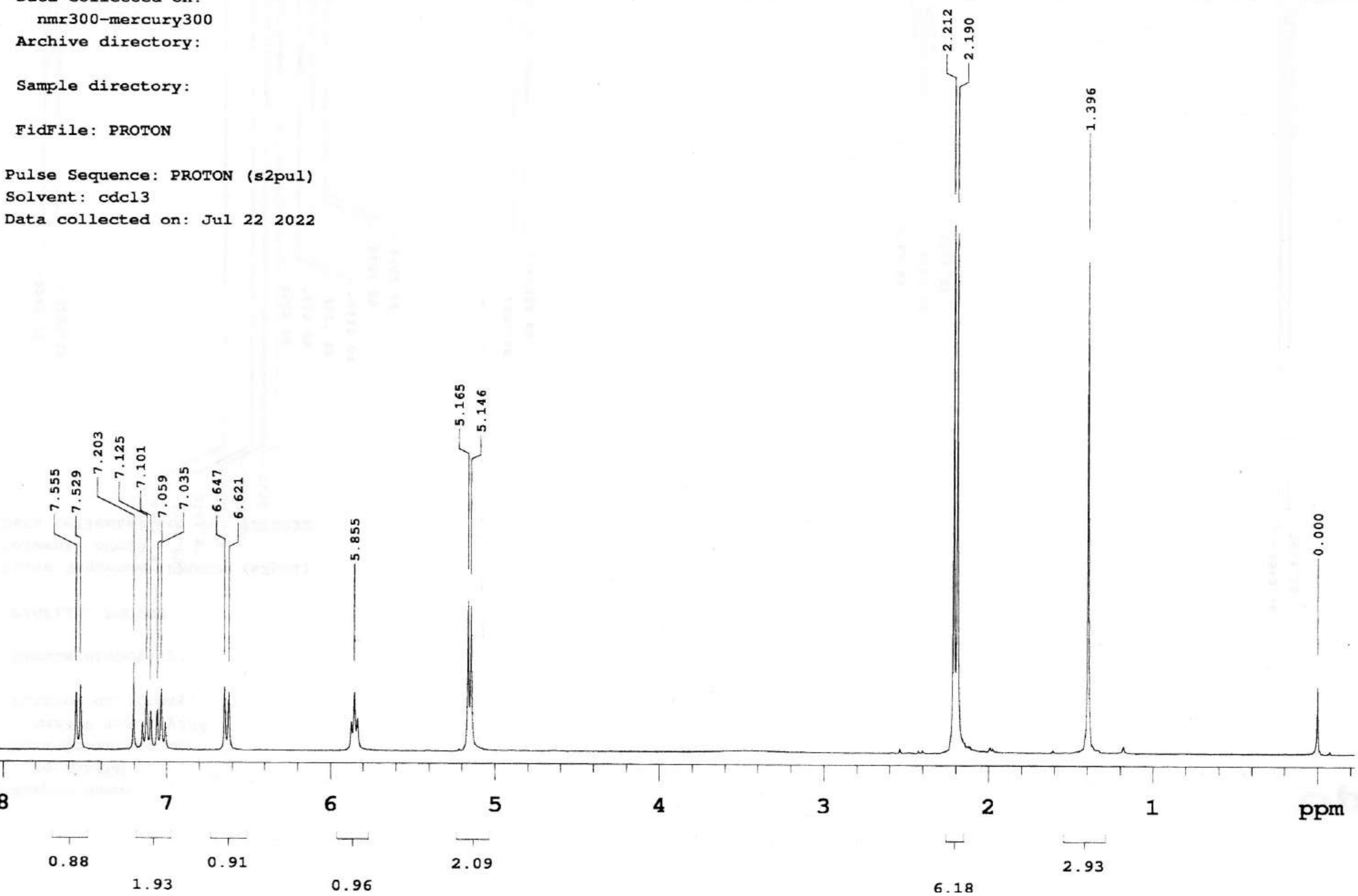

Sample Name:
5g-ZALARU
Data Collected on:
nmr300-mercury300
Archive directory:

Sample directory:

FidFile: PROTON

Pulse Sequence: PROTON (s2pul)
Solvent: cdcl3
Data collected on: Jul 22 2022

OpenVnmrJ

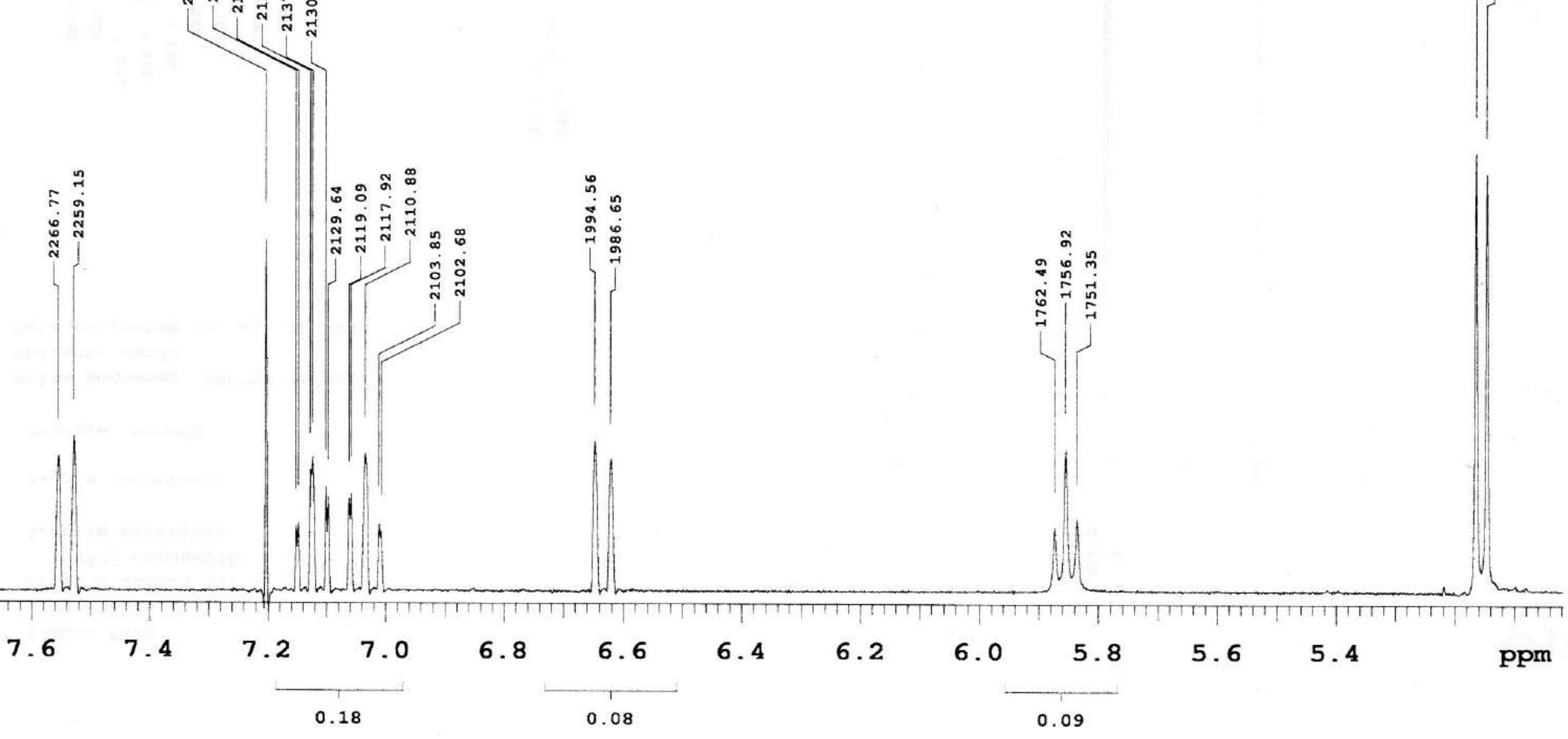

Supplement: Supplementary file 1 [file antibiotics-11-01094-s001.zip › antibiotics-1826953-supplementary.pdf]
